# Detection of *Anaplasma phagocytophilum*, *Babesia odocoilei*, *Babesia* sp., *Borrelia burgdorferi* Sensu Lato, and *Hepatozoon canis* in *Ixodes scapularis* Ticks Collected in Eastern Canada

**DOI:** 10.3390/pathogens10101265

**Published:** 2021-10-01

**Authors:** John D. Scott, Risa R. Pesapane

**Affiliations:** 1 Upper Grand Tick Centre, 365 St. David Street South, Fergus, ON N1M 2L7, Canada; 2 Department of Veterinary Preventive Medicine, College of Veterinary Medicine, The Ohio State University, Columbus, OH 43210, USA; pesapane.1@osu.edu; 3 School of Environment and Natural Resources, College of Food, Agricultural, and Environmental Sciences, The Ohio State University, Columbus, OH 43210, USA

**Keywords:** tick-borne pathogens, *Anaplasma phagocytophilum*, *Borrelia burgdorferi* sensu lato, *Babesia odocoilei*, *Babesia* sp., *Hepatozoon canis*, blacklegged tick, *Ixodes scapularis*, Ontario, Quebec

## Abstract

Tick-borne pathogens cause infectious diseases that inflict much societal and financial hardship worldwide. Blacklegged ticks, *Ixodes scapularis*, are primary vectors of several epizootic and zoonotic pathogens. The aim was to find varius pathogens of *I. scapularis* and to determine their prevalence. In Ontario and Quebec, 113 *I. scapularis* ticks were collected from songbirds, mammals, including humans, and by flagging. PCR and DNA sequencing detected five different microorganisms: *Anaplasma phagocytophilum*, 1 (0.9%); *Babesia odocoilei*, 17 (15.3%); *Babesia microti*-like sp., 1 (0.9%); *Borrelia burgdorferi* sensu lato (Bbsl), 29 (26.1%); and *Hepatozoon canis*, 1 (0.9%). Five coinfections of Bbsl and *Babesia odocoilei* occurred. Notably, *H. canis* was documented for the first time in Canada and, at the same time, demonstrates the first transstadial passage of *H. canis* in *I. scapularis.* Transstadial passage of Bbsl and *B. odocoilei* was also witnessed. A novel undescribed piroplasm (*Babesia microti*-like) was detected. An established population of *I. scapularis* ticks was detected at Ste-Anne-de-Bellevue, Quebec. Because songbirds widely disperse *I. scapularis* larvae and nymphs, exposure in an endemic area is not required to contract tick-borne zoonoses. Based on the diversity of zoonotic pathogens in *I. scapularis* ticks, clinicians need to be aware that people who are bitten by *I. scapularis* ticks may require select antimicrobial regimens.

## 1. Introduction

Tick-borne zoonotic diseases cause unrelenting woe and hardship worldwide. Polymicrobial infections typically complicate diagnosis and augment disease severity, which result in more disabling sequelae of illness. Tick-borne polymicrobial infections are relatively common in patients, but infrequently reported [1,2,3]. These zoonoses range from subclinical to fatal infections with a disproportionate incidence in children and the elderly.

In North America, Lyme disease caused by *Borrelia burgdorferi* sensu lato (Bbsl) is the most commonly reported tick-borne disease. Several other tick-related diseases include human anaplasmosis, human babesiosis, and human bartonellosis. When ticks are laden with multiple pathogens, they have the potential for simultaneous transmission of two or more pathogenic microorganisms to a host [4,5]. Notably, four different tick-borne zoonotic pathogens in a single blacklegged tick, *Ixodes scapularis* (Acari: Ixodidae), have been reported [6].

In North America, *I. scapularis* can harbor any combination of at least nine different pathogens [7,8]. This tick species is widely distributed east of the Rocky Mountains, and is dispersed by migratory songbirds (Order: Passeriformes) as far west and north as northwestern Alberta, and as far east and north as the southern part of Newfoundland and Labrador [9]. Passerine migrants play a key role in the wide dispersal of *I. scapularis* larvae and nymphs especially during northward spring migration [8,9,10,11,12,13,14]. In eastern and central Canada, songbird-transported *I. scapularis* ticks may be infected with a plethora of tick-borne pathogens, especially Bbsl and *Babesia* *odocoilei* (Apicomplexa: Piroplasmida: Babesiidae). Of note, a triple co-infection of Bbsl, *Babesia microti*, and *A. phagocytophilum* has been documented in a blacklegged tick nymph parasitizing a Veery [15].

Recently, molecular and biomedical researchers revealed that human babesiosis caused by *B. odocoilei* is pathogenic [16]. Not only does this tick-borne zoonotic pathogen infect humans, this *Babesia* sp. infects a wide range of cervid and bovid hosts. The biographical distribution of *B. odocoilei* coincides with *I. scapularis*, which are indigenous east of the Rocky Mountains [17,18,19], and in far-western North America, the western blacklegged tick, *Ixodes pacificus* [20], is indigenous. Pertinent to *I. scapularis* ticks, *B. odocoilei* has transovarial transmission (female to eggs to larvae) [19,21] and, similarly, transstadial passage (larva to nymph or nymph to adult) [8,14,19,21]. The present epidemiological study, which is centered directly in the territorial heartland of *I. scapularis* and white-tailed deer, *Odocoileus virginianus*, was conducted in the Great Lakes basin [22,23,24].

The aim of this tick study was to (1) assess the geographic distribution of tick-borne pathogens in eastern Canada, (2) ascertain the presence of zoonotic pathogens in field-collected ticks, and (3) determine the prevalence of select pathogens in *I. scapularis*. This newfound study elucidates the first report of *B. odocoilei* in eastern Ontario and the *Hepatozoon canis* piroplasm in Canada.

## 2. Results

### 2.1. Tick Collection

**Figure 1:**
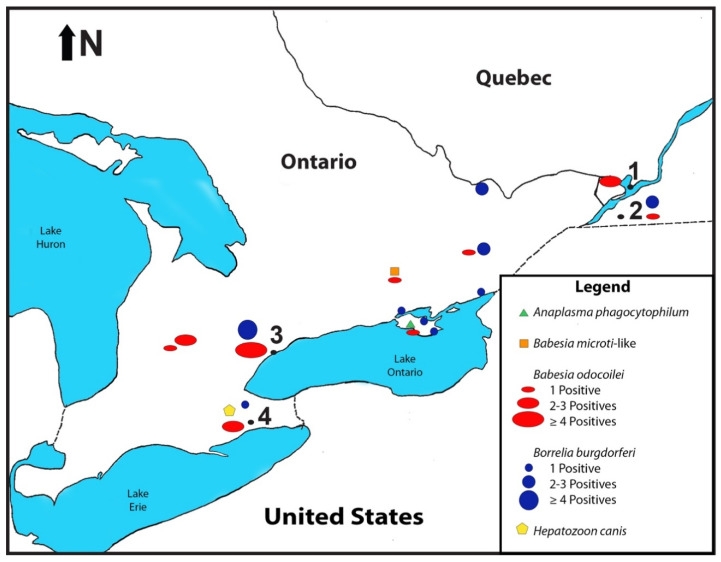
Map showing core geographic locations of *Ixodes scapularis* collected. (**1**) McGill Bird Observatory, Ste-Anne-de-Bellevue, Quebec: 45.43 N, 73.94 W. (**2**) Montée Biggar, Quebec; 45.09 N, 74.22 W. (**3**) Rouge National Urban Park, Toronto, Ontario; 43.82 N, 79.17 W. (**4**) Ruthven Park National Historic Site Banding Station, Haldimand Bird Observatory, Cayuga, Ontario; 42.97 N, 79.87 W.

A total of 113 *I. scapularis* ticks were collected from 69 vertebrate hosts (songbirds, mammals including humans) and by flagging (Table 1, Figure 1). These collections comprised of 23 songbirds (n = 29 ticks), 46 mammals (n = 59 ticks), and flagging (n = 25 ticks) (Table 1). Two ticks collected from songbirds were lost during field operations, and 111 ticks were tested for tick-borne zoonotic pathogens.

### 2.2. Pathogen Detection

Overall, PCR and DNA sequencing found pathogen targets in 49/111 (44.1%) of *I. scapularis* ticks tested. Based on the five microbial targets, 1/111 (0.9%) *I. scapularis* tested positive for *A. phagocytophilium*; 1/111 (0.9%) positive for *B. microti*-like sp.; 17/111 (15.3%) were positive for *B. odocoilei*; 29/111 (26.1%) positive for Bbsl; and 1/111 (0.9%) positive for *H. canis* (Table 2). 

Fourteen (56%) of 25 *I. scapularis* adults collected by flagging were positive for Bbsl. Five (20%) of these 25 *I. scapularis* adults were infected with *B. odocoilei*. Four of these females were co-infected with Bbsl *and B. odocoilei*. All ticks in this study were negative for *B. microti*.

Of special enzootic note, a fully engorged *I. scapularis* nymph was collected from a House Wren on 19 May 2020 at Ruthven Park, Cayuga, Ontario. The nymph underwent transstadial passage, and molted to a male in 42 days (Table 1). By using PCR testing, DNA sequencing, and Basic Local Alignment Search Tool (BLAST) analysis, *Hepatozoon canis* was detected. This detection is the first report of *H. canis* in an *I. scapularis* tick, and constitutes the first detection of *H. canis* in Canada.

An engorged *I. scapularis* female was collected from a cat in Picton, Ontario, on 16 October 2020, and this partially fed female tested positive for *A. phagocytophilum*. This is the first detection in Canada of *A. phagocytophilum*-infected *I. scapularis* tick parasitizing a domestic cat. 

Five *I. scapularis* females were collected from a North American porcupine, *Erethizon dorsatum*, hit by a moving vehicle at Westboro, Ontario, on 17 October 2020. Three females were infected with Bbsl, and one female was infected with *B. odocoilei*. One of these females was co-infected with Bbsl and *B. odocoilei*.

Six (30%) of 20 *I. scapularis* nymphs collected from passerine birds were positive for *B. odocoilei*. 

## 3. Discussion

Our findings reveal a quaint observation about the incidence of tick-borne pathogens in eastern Canada. Based on our results, babesial infections are more common on songbird-transported *I. scapularis* ticks than the Lyme disease bacterium. Although emergency departments and medical clinics are targeting tick bites with short-term antibiotic treatments for Lyme disease, they are missing the fact that anti-*Babesia* therapy may be required. Other tick-borne pathogens, such *A. phagocytophilum* and *H. canis,* are also entering the pathogenic picture.

### 3.1. Pathogen-Positive I. scapularis

**Figure 2:**
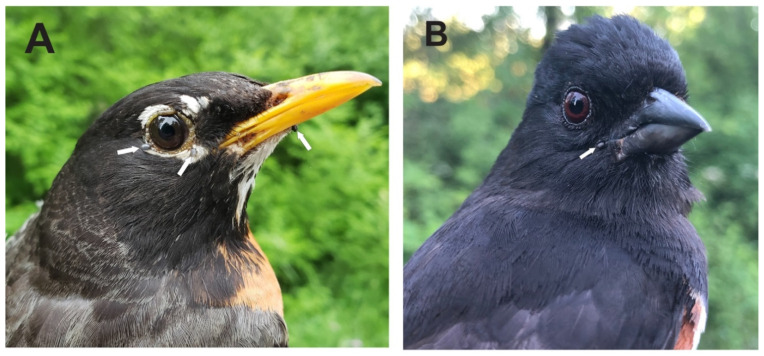
Ground-frequenting songbirds parasitized by *Borrelia burgdorferi* sensu lato-positive larval and nymphal blacklegged ticks: (**A**) American Robin, adult breeding male, and (**B**) Eastern Towhee, second year male. These songbird-derived ticks were collected during the nesting season. Photo credits: Ana Morales.

When we consider all mobile life stages (larvae, nymphs, adults) of 111 *I. scapularis* ticks tested, we obtained pathogen-positive ticks comprising Bbsl, 29 (26.1%); *B. odocoilei*, 17 (15.3%); *B. microti*-like sp., 1 (0.9%); *A. phagocytophilum*, 1 (0.9%); and *H. canis*, 1 (0.9%). Tick researchers in the Great Lakes basin have likewise detected Bbsl, *Babesia* spp., and *A. phagocytophilum* in *I. scapularis* ticks [22,23,24]. Within the the same basin, a research team found an infection prevalence of 71% for *B. odocoilei* detected in *I. scapularis* females collected from cats and dogs in west-central Ontario [25]. Even though the Bbsl infection prevalence for *I. scapularis* was lower than *B. odocoilei* for songbird-derived ticks (Figure 2), we found that it was highest overall for *I. scapularis* parasitizing avifauna(Table 1). Notably, five (4.5%) of 111 *I. scapularis* adults in this study were co-infected with Bbsl and *B. odocoilei*. Whenever people are bitten by a *I. scapularis* tick, clinicians must realize that patients may require a combination of antimicrobials to match the pathogen load.

### 3.2. Distribution of B. odocoilei

Both *I. scapularis* ticks and white-tailed deer have a wide distribution east of the Rocky Mountains, and play a key role in the enzootic transmission cycle for *B. odocoilei*. Continent-wide, *B. odocoilei* has been documented in several provinces and states including Texas [19,26], California [20], Oklahoma [26], Virginia [27], Pennsylvania [13,24,27,28], New York state [29], Michigan [22], Wisconsin [23], Indiana [23], Maine [30], Ontario [8,25], Quebec [8], and Saskatchewan [31]. Consistent with these research findings, we also located a new population of *I. scapularis* ticks in a western suburb of Montreal, Quebec.

### 3.3. Comparative Relationship between Babesia Spp.

Many people in the medical field contend that *B. duncani* and *B. microti* are unequivocally the main cause of human babesiosis in North America [32,33]. However, neither *B. duncani* nor *B. microti* were detected during the present study. Instead, *B. odocoilei* was the focal *Babesia* species. Of medical significance, *B. odocoilei* causes human babesiosis and is pathogenic to humans [16]. Throughout the present study, there was only one *B. microti*-like piroplasm; it was detected in an *I. scapularis* female removed from a cat. In the Great Lakes basin, white-tailed deer are the reservoir of *B. odocoilei*, and *I. scapularis* is the vector [25]. Both are indigenous throughout the Great Lakes region, and perpetuate an enzootic transmission cycle of *B. odocoilei*.

### 3.4. Source of B. odocoilei-Infected Ticks

**Figure 3:**
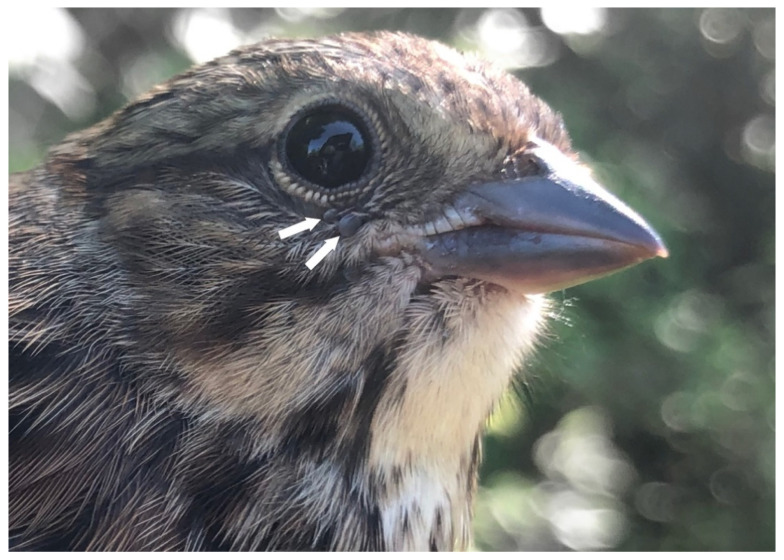
Song Sparrow, hatch year, parasitized by two *Ixodes scapularis* nymphs of which one was positive for *Babesia odocoilei*. These ticks were collected during the nesting period. Photo credits: Ana Morales.

The source of *I. scapularis* parasitizing songbirds varies depending on the time of year. Throughout the temperate months, 50% of the *B. odocoilei*-infected *I. scapularis* were collected during northward spring migration, whereas 17% were obtained during the nesting and fledgling period at Montée Biggar, Quebec, in juxtaposition to the nesting spot (Figure 3). Of significance, 33% of the *B. odocoilei*-infected *I. scapularis* were attained during fall migration. Ticks acquired during nesting will account for the locally acquired ticks [34], whereas infestations on neotropical passerines, which are heading to the boreal forest to breed and rear their young [13], will represent a large portion of the ticks occurring from long-distant dispersal.

### 3.5. Pathways of B. odocoilei

The origin of *B. odocoilei* detected in the *I. scapularis* larva, which was collected from a Common Yellowthroat, is uncertain. The larva may have become infected via transovarial transmission or, alternatively, from the host bird. To date, there is no conclusive evidence to show that songbirds are reservoir-competent hosts of *B. odocoilei*.

It is noteworthy that transovarial transmission of *B. odocoilei* has been documented in *I. scapularis* [19,21,26]. Because the *I. scapularis* larva, which was collected from the Common Yellowthroat, successfully molted to a nymph in 29 days, we affirm that transstadial passage of *B. odocoilei* occurs in *I. scapularis*. Transstadial passage of *B. odocoilei* in collections in 2018 and 2019 from larva to nymph [8,14,34], and also from nymph to adult, revealed transstadial passage [8,14,34]. Since transovarial transmission and transstadial passage occur in *I. scapularis* [19,26], these pathways provide a continuous cycle of *B. odocoilei* infection. Notably, southeast Texas (the original site of *B. odocoilei* study) and southwest Quebec are 15 latitudes apart. 

Tick researchers recently reported the coexistence of Bbsl and *B. odocoilei* in an *I. scapularis* nymph collected in 2018 from a Veery in Canada [13]. In the present study, co-infection of Bbsl and *B. odocoilei* has also been documented in *I. scapularis* collected from avian and mammalian hosts and, likewise, by flagging. 

### 3.6. Phylogeny of B. microti-Like Piroplasm

The phylogeny of a *B. microti*-like piroplasm detected in an *I. scapularis* female is currently an undescribed species. Based on BLAST analysis, supported with 99% coverage, the *Babesia* variant has a sequence divergence of 95.7% relative to the type strain of *B. microti*. The host cat may have been infected with this variant at the time of tick bite and passed the infection during the blood meal. Alternatively, because the *I. scapularis* female had imbibed two previous blood meals prior to the blood meal from a suitable host, this tick may have become infected during the larval-nymphal blood meal or the nymphal-adult blood meal. This *Babesia* sp. variant could be a newly discovered species that is yet to be identified. All the other babesial infections in this pathogen study were *B. odocoilei*. Of note, *B. microti* was not detected in any of the *I. scapularis* ticks collected during this study.

### 3.7. Ixodes scapularis Parasitize Mammalian Hosts

In the present study, a North American porcupine was infested with five *I. scapularis* ticks. Three of these females were infected with Bbsl, and one of these partially engorged females was infected with *B. odocoilei*. Previously, *B. odocoilei* was detected in *I. scapularis* adults collected from canids and felids in the Huronia area of southern Ontario [25] and, in this sylvan environment, this tick species exhibited a significantly higher prevalence (71%) of *B. odocoilei* [25]. This infestation signifies the first documentation of a mammalian parasitism in Canada where the *I. scapularis* was co-infected with Bbsl and *B. odocoilei*. North American porcupines are denizens of arboreal habitats, and help to propel the enzootic transmission cycle of Bbsl and *B. odocoilei*. Whenever patients are bitten by *I. scapularis* ticks, each one needs to be tested for tick-borne pathogens, especially Bbsl and *B. odocoilei*. For efficacious treatment, an anti-babesial regimen should be considered for this type of polymicrobial infection.

### 3.8. Novel Discovery of H. canis in Canada

The detection of *H. canis* in a tick in Canada is a newfound discovery. Moreover, this is the first time that *H. canis* has been detected in an *I. scapularis* tick. Neotropical songbirds transport ticks into Canada from as far south as the northern part of South America [8,9,10,11,12,13,14], and would most likely acquire this *H. canis*-infected nymph in neotropical regions or the southern United States. House Wrens, *Troglodytes aedon*, have southern breeding grounds in Central America and South America, as far south as Argentina, and commonly have hemisphere-spanning migration. The House Wren likely made a migratory stopover in the southern United States, and transported the *H. canis*-infected *I. scapularis* nymph firsthand via cross-border flight to Ontario. By allowing this fully engorged nymph to molt to an adult (male), we were able to show that *H. canis* successfully undergoes transstadial passage. Therefore, this alveolate can successfully pass from larva to nymph and, likewise, from nymph to adult.

More than 300 species of *Hepatozoon*, which belong to the family Hepatozoidae, are known to infect animals [35]. *Hepatozoon canis* is typically found in the brown dog tick, *Rhipicephalus sanguineus*, which has wide distribution, including southern Europe, Africa, and Asia.

Members of the genus *Hepatozoon* are well known in veterinary circles for causing tick-borne diseases, namely hepatozoonosis in certain mammals. *Hepatozoon canis*, a one-celled, intraerythrocytic parasite infects members of the family Canidae (e.g., dog, coyotes, and foxes) [35]. Biogeographically, *H. canis* has been reported in several states across the southern United States [36]. It is noteworthy that *H. canis* is epizootic in dogs, and clinical manifestations include lethargy, fever, weight loss, decreased appetite, muscle pain/weakness, reluctance to move, and discharge from eyes and nose and, in severe cases, it can cause death [36].

In the United States, American canine hepatozoonosis is also caused by *Hepatozoon americanum*, and is delineated from *H. canis* by molecular identification. When dogs are bitten by or ingest Gulf Coast ticks, *Amblyomma maculatum*, which is the source of infective oocysts of *H. americanum*, the host canid normally becomes infected [35].

### 3.9. American Robins Are Competent Reservoirs of B. burgdorferi Sensu Lato

In the present study, two *I. scapularis* larvae and one *I. scapularis* nymph were collected from an American Robin, and a single larva molted to a nymph in 27 d; it was positive for Bbsl (Figure 1). This event shows transstadial passage of Bbsl in *I. scapularis*. Both Bbsl and *B. odocoilei* are maintained in the tick midgut during the molt [37], and both of these pathogenic microorganisms successfully retain their viability to pursue host-seeking activities. 

Since the larva had not had a previous blood meal prior to feeding on the American Robin, we re-iterate the fact that American Robins are reservoir-competent hosts. Using spirochete-free, xenodiagnostic larvae, researchers reported that the American Robin is a reservoir host of Bbsl [38]. Because transovarial transmission of Bbsl is not present in *I. scapularis*, we confirm that the American Robin is a reservoir-competent host of Bbsl.

### 3.10. Human Babesiosis as a Tick-Borne Zoonosis

Human babesiosis has received considerable attention as a tick-borne zoonosis worldwide. Historically, this single-celled intraerythrocytic piroplasmid was initially discovered in 1888 by Victor Babes, a Romanian pathologist [39]. Five years later, American researchers found that ticks are the primary vector that transmit *Babesia* spp. to terrestrial vertebrate [40]. The first documentation of a human case of babesiosis was described in a splenectomized Croatian cattle farmer in 1956 [41], and the red blood cell parasite was later named *Babesia divergens*. Clinical manifestations relating to human babesiosis vary from asymptomatic to life-threatening. Symptoms are wide-ranging, including profound fatigue, flushing, sweats (especially night sweats), chills, muscle aches, increased thirst, headaches, sleep disturbance, fever, and anxiety. 

Human babesiosis caused by *B. odocoilei* is a recalcitrant infection [16]. Human subjects often have low parasitemia [16] but are symptomatic. When *B. odocoilei*-infected red blood cells adhere to fibrin, which line the endothelial cells of capillaries and venules, cytoadherence (binding to endothelial cells) and sequestration result. This fibrin-bonding mechanism is similar to the sequestration discerned with *B. canis* in dogs [42]. Cytoadherence and sequestration develop as the microvasculature of capillaries and venules fill up with infected erythrocytes. Longstanding infections of *B. odocoilei* can induce clinical symptoms involving ischemia, profound fatigue, severe hemolysis, thrombocytopenia, dysautonomia, hemodynamic instability, sustained inflammation, and multi-organ dysfunction and, eventually, may lead to death [32,33,43]. A fibrin-dissolving enzyme, such as lumbrokinase, is required to slowly loosen the infected piroplasmids. The procedure is a gradual, long-term anti-babesial therapy. Based on fibrin-bonding sequestration, *B. odocoilei* ranks as the most pronounced chronic human babesiosis in North America. 

A wide diversity of *Babesia* species that cause human babesiosis globally include *B. crassa, B. divergens*, *B. duncani* (WA1)*, B. microti, B. odocoilei, B. venatorum,* *Babesia* sp. variants CA1, CA3, and CA4, *Babesia* sp. CN1, *Babesia divergens*-like MO1, and *Babesia* sp. TW1 [16,44,45,46,47,48,49,50,51,52]. 

Not only are babesial piroplasmids transmitted by ticks, they are transmitted by blood transfusion [53], organ transplantation [54], and maternal–fetal transmission [55,56,57,58]. Similarly, Lyme disease spirochetes can be transmitted by maternal–fetal transmission [59,60,61,62,63]. Throughout a person’s life, these two pathogens can be an insidious life-altering disease. In reality, co-infection of *B. odocoilei* and Bbsl can potentially occur in the neonate if a pregnant person is bitten by an *I. scapularis* tick that is coinfected with these two tick-borne, zoonotic microorganisms.

### 3.11. Endemic Area Not Required to Contract Tick-Borne Diseases

Even though people are bitten in endemic areas, our results show that people can be bitten anywhere that ticks drop from songbirds and mammals. Passerine migrants are directly involved in the wide dispersal of pathogen-positive *I. scapularis* ticks [8,9,10,11,12,13,14]. These epizootic and zoonotic pathogens can be carried hundreds of kilometers and dispersed across the landscape [8,9,10,11,12,13,14]. Based on our findings at Montée Biggar, Quebec (Table 1, Figure 1), we re-affirm that this nesting site is an endemic area for *I. scapularis* [34]. Both *I. scapularis* larvae and nymphs were collected from nesting songbirds, which constitutes an established population [34,64]. In addition, white-tailed deer are prominent in this locale, and support the reproduction and propagation of *I. scapularis*, especially adults. Outdoor people can be bitten whenever they venture into grassy and woodland areas. At the time of fall migration, songbirds fly southward from this nesting and fledgling site, and widely disperse larval and nymphal *I. scapularis* while en route to their southern breeding grounds. Since songbirds widely disperse songbird-transported ticks during bi-directional flight, people do not have to reside in an endemic area to acquire tick-borne zoonotic diseases.

In 2020, six (86%) of seven songbird-transported *I. scapularis*, which were held to molt, successfully developed to the next developmental life stage. These ecological findings help to substantiate that an established population of *I. scapularis* is located at Ste-Anne-de-Bellevue, Quebec. This western Montréal suburb at the junction of the Ottawa River and the St. Lawrence River fulfil the criteria (two life stages in a collection period) for an established population of *I. scapularis* ticks [34,64]. Based on these tick collections, this campus woods represents an established population of *I. scapularis* [34,64] and, additionally, has an enzootic transmission cycle for both Bbsl and *B. odocoilei*. 

### 3.12. Diversity of Pathogens in I. scapularis

When bitten by a questing *I. scapularis* tick, people can contract any one of at least nine different tick-borne zoonotic pathogens [7,16,25]. We detected Bbsl (the causal organism of Lyme disease) and *B. odocoilei* (the causal organism of human babesiosis) in both larval and nymphal *I. scapularis*. 

When it comes to tick bites, the present mindset focuses on Lyme disease. In the present study, 16.2% of the *I. scapularis* ticks tested were infected with *Babesia* spp. Since *Babesia* spp. require a different treatment protocol than other pathogenic microorganisms, healthcare providers need to be aware that people bitten by an *I. scapularis* tick may be infected with *B. odocoilei*. Medically, *B. odocoilei* causes human babesiosis and is pathogenic to humans [16]. Since cross-reactivity is an ongoing issue with *Babesia* spp., other modes of testing, such as molecular identification (e.g., PCR, DNA sequencing), are needed to firmly delineate *Babesia* species [65]. When it comes to *I. scapularis* tick bites, clinicians must consider a wide spectrum of pathogens and include them in the differential diagnosis. Consideration of *Babesia* piroplasmids is vitally important in administering an efficacious approach to treatment.

## 4. Materials and Methods

### 4.1. Tick Collection

Ticks were collected from songbirds by bird banders using super-fine point stainless steel forceps. These ticks were put into 8.5 mL, polypropylene, round-bottom tubes, 75 × 15 mm containing a slightly moistened paper towel. After inserting ticks, the mouth of the tube was covered with a piece of tulle netting, and a push-cap with a 7 mm hole was placed into the mouth of the tube to prevent live ticks from escaping. The collection tube was put into a self-sealing plastic bag with a slightly moistened paper towel. An attached label for the background information of each bird was completed. Live ticks were sent directly to the lab (J.D.S.) for identification. Partially and fully engorged larvae and nymphs were held to molt to the next developmental life stage using a photoperiod of 16:8 h (Light: Dark) with ambient temperature and 90–95% humidity. As the daylength shortened, a 12W, full-spectrum LED daylight bulb (LifeEnergy Systems, Canada) was used, and plugged into an electrical timer. Complete tick records (i.e., geographic location, collecting date, tick species, developmental life stage, and bird species) were logged for each tick collection.

Ticks from humans were self-collected, put in a tightly sealed vial containing 99% isopropyl alcohol, and promptly submitted to the laboratory (J.D.S.) for identification. These vials were then sent to the laboratory (R.R.P.) by courier for pathogen testing.

Ticks parasitizing mammals were collected by veterinarians and veterinarian technicians using fine-pointed stainless steel forceps, and then placed directly in 2 mL micro tubes containing 94% ethyl alcohol. At the end of the collection period for *I. scapularis* adults, all ticks were sent to the lab (J.D.S.) for identification. All collected ticks were identified morphologically to species, life stage, and gender (if adult) by using a stereoscopic microscope and taxonomic keys [66,67,68].

A flannel-back vinyl cloth measuring 59 cm × 71 cm attached to an aluminum telescopic window washer’s pole, 197 cm in length, was used to flag for questing *I. scapularis* adults. Flannel-back vinyl cloth was used because it does not blow around in the wind. In addition, it withstands thorny stems of brambles, a cane-like, prickly shrub. When low-lying vegetation is dry to the touch, flagging is conducted between 1000 hrs. and 1600 hrs. during cloudy or partly cloudy days. Flagging was done in grassy areas and, similarly, along trails and pathways. 

### 4.2. Pathogen Detection

Ethanol storage medium was allowed to evaporate before proceeding with tick DNA extraction. Clean razor blades were used to bisect each tick (anteriorly to posteriorly through the capitulum) to isolate a 5–10 mg fragment [69]. DNA was extracted using the Purelink Genomic DNA Mini Kit (Invitrogen, Waltham, MA, USA), following manufacturer’s instructions, and eluted in 80 µL Genomic Elution Buffer. The quantity and quality of DNA was assessed using the Nanodrop One spectrophotometer (Thermo Scientific, Waltham, MA, USA).

Real-time PCR amplification was performed in duplicate aliquots using 2 µL of extracted DNA in a 20 µL reaction of Taqman Fast Advanced Master Mix (Applied Biosystems, Waltham, MA, USA). As well, the published primers and probes for the 16S gene of Bbsl [70] and *msp2* gene of *A. phagocytophilum* [71] were employed. The *B. burgdorferi* s.l. probe specifically excluded *Borrelia miyamotoi* [70]. A sample was considered positive when the cycle threshold (CT) was less than 40 with a characteristic curve. Conventional PCR amplification for the 18S gene of *Babesia* spp. was performed in duplicate and visualized on a 1% gel using methods and reagents as previously described [8]. Samples were considered positive when amplicons of 400–500 base pairs (bp) were observed. All PCR reactions included molecular-grade water and, also, synthetic gBlock gene fragments (Integrated DNA Technologies, Coralville, IA, USA) of *B. burgdorferi* (MH781147.1), *A. phagocytophilum* (AY151054.1), and *B. microti* (MT974173.1) as controls. When tick species was uncertain, based on morphology alone, the 16S and 12S genes were amplified using 2 µL of extracted DNA in a 25 µL conventional PCR reaction of GoTaqGreen Master Mix (Promega, Madison, WI, USA) with earlier established primers [72,73], followed by sequencing. 

PCR products were prepared for sequencing using either ExoSAP-IT (Applied Biosystems) or PureLink Quick Gel Extraction Kit (Invitrogen, Waltham, MA, USA). All sequencing was performed at the Genomics Shared Resource laboratory at the Comprehensive Cancer Center within the Ohio State University using forward and reverse primers. Manual edits and alignments were performed in the program CLC Main Workbench v.21.0.3 (www.qiagenbioinformatics.com/, accessed on 27 August 2021) followed by BLAST in GenBank (NCBI; http://blast.ncbi.nlm.nih.gov/Blast.cgi, accessed on 27 August 2021).

## 5. Conclusions

In this tick-host-pathogen study, we crossed a new threshold with the novel discovery of *H. canis* in Canada. Moreover, we have disclosed the premiere detection of *H. canis* in an *I. scapularis* tick. Since this *I. scapularis* nymph was collected during peak spring migration, this bird parasitism by a neotropical songbird documents cross-border flight into Canada. We provide the first authentic documentation of an *I. scapularis* tick infected with *A. phagocytophilum* parasitizing a feline host in Canada. Based on DNA sequencing and BLAST analysis, we document the first *B. microti*-like pathogen in Canada and, upon further multi-locus analysis, could potentially be a new *Babesia* sp. Overall, 15.3% of *I. scapularis* were infected with *B. odocoilei* in eastern Canada, and similarly, 30% of the songbird-transported *I. scapularis* nymphs were positive for *B. odocoilei.* Each of these babesial infection groupings present a significant health risk. Because migratory songbirds widely disperse *I. scapularis* ticks, people do not have to frequent an endemic area to contract tick-borne zoonoses. When treating *I. scapularis* tick bites, clinicians at medical clinics and emergency departments need to be aware that there is a subtle difference between antimicrobial therapies for *B. odocoilei* and Bbsl.

## Figures and Tables

**Table 1:** Detection of tick-borne pathogens in *Ixodes scapularis* ticks collected from vertebrates and by flagging, Ontario and Quebec, 2020.

Source	No. of Hosts	No. Ticks Collected	*Ixodes scapularis*				Ticks Tested	Pathogens			
			L	N	M	F		Bbsl	Bab	Aph	Hcn
**Songbirds**											
Lincoln’s Sparrow, *Melospiza lincolnii*	1	1	0	1	0	0	1	0	0	0	0
Indigo Bunting, *Passerina cyanea*	1	1	0	1	0	0	1	0	0	0	0
House Wren, *Troglodytes aedon*	4	5	0	5	0	0	5	1	0	0	1
Common Yellowthroat, *Geothlypis trichas*	5	5	3	2	0	0	5	0	3	0	0
Veery, *Catharus fuscescens*	3	3	0	3	0	0	3	0	0	0	0
Baltimore Oriole, *Icterus galbula*	2	2	0	2	0	0	2	0	1	0	0
American Robin, *Turdus migratorius*	1	3	1	1	0	0	2	1	0	0	0
Song Sparrow, *Melospiza melodia*	2	4	0	3	0	0	3	1	1	0	0
Black-capped Chickadee *Poecile atricapillus*	1	1	0	1	0	0	1	0	0	0	0
Eastern Towhee, *Pipilo erythrophthalmus*	1	1	0	1	0	0	1	1	0	0	0
Ovenbird, *Seiurus aurocapilla*	1	2	2	0	0	0	2	0	0	0	0
Northern Waterthrush, *Parkesia noveboracensis*	1	1	1	0	0	0	0	1	1	0	0
**Mammals**											
Dog, *Canis familiaris*	32	39	0	0	4	35	39	5	3	0	0
Cat, *Felis catus*	9	11	0	0	2	9	11	1	3	1	0
North American porcupine, *Erethizon dorsatum*	1	5	0	0	0	5	5	3	1	0	0
Human, *Homo sapiens*	4	4	0	0	0	4	4	2	0	0	0
**Flagging**											
Vegetation	0	25	0	0	8	17	25	14	5	0	0
Total	69	113	7	20	14	70	111	29	18	1	1

L, larva(e); N, nymph(s); M, male(s); F, female(s). Bbsl, *Borrelia burgdorferi* sensu lato; Bab, *Babesia* spp.; Aph, *Anaplasma phagocytophilum*; Hcn, *Hepatozoon canis*.

**Table 2:** Dataset denoting representative apicomplexan microorganisms detected in *Ixodes scapularis* collected from vertebrates in Ontario and Quebec, 2020. L, larva(e); N, nymph(s); M, male(s); F, female(s).

Geographic Location.	Source	Life Stage	Pathogen	GenBank Accession Number
Cayuga, ON	Common Yellowthroat	N	*B. odocoilei*	MZ841618
Belwood, ON	Cat, domestic	F	*B. odocoilei*	MZ841619
Montée Biggar, QC	Song Sparrow	N	*B. odocoilei*	OK041475
Ste-Anne-de-Belleville, QC	Common Yellowthroat	L	*B. odocoilei*	MZ841620
Toronto, ON	Vegetation	M	*B. odocoilei*	MZ841621
Fergus, ON	Dog, domestic	F	*B. odocoilei*	MZ841622
Westport, ON	Porcupine	F	*B. odocoilei*	MZ841623
Bloomfield, ON	Dog, domestic	F	*B. odocoilei*	MZ841624
Tweed, ON	Cat, domestic	F	*B. microti*-like	MZ841625
Tweed, ON	Dog, domestic	F	*B. odocoilei*	OK041476
Cayuga, ON	House Wren	N	*H. canis*	MZ841626

## Data Availability

All data are reported in the manuscript.

## References

[1] Magnarelli L.A., Dumler J.S., Anderson J.F., Johnson R.C., Fikrig E. (1995). Coexistence of antibodies to tick-borne pathogens of babesiosis, ehrlichiosis, and Lyme borreliosis in human sera. J. Clin. Microbiol..

[2] Mitchell P.D., Reed K.D., Hofkes J.M. (1996). Immunoserologic evidence of coinfection with *Borrelia burgdorferi*, *Babesia microti*, and human granulocytic *Ehrlichia* species in residents of Wisconsin and Minnesota. J. Clin. Microbiol..

[3] Stricker R.B. (2003). Lyme disease: A potential polymicrobial infection. ASM News.

[4] Benach J.L., Coleman J.L., Habicht G.S., MacDonald A., Grunwaldt E., Giron J.A. (1985). Serological evidence for simultaneous occurrences of Lyme disease and babesiosis. J. Infect. Dis..

[5] Anderson J.F., Mintz E.D., Gadbaw J.J., Magnarelli L.A. (1991). *Babesia microti*, human babesiosis, and *Borrelia burgdorferi* in Connecticut. J. Clin. Microbiol..

[6] Sanchez-Vicente S., Tagliafierro T., Coleman J.L., Benach J.L., Tokarz R. (2019). Polymicrobial nature of tick-borne diseases. mBio.

[7] Nicholson W.A., Sonenshine D.E., Noden B.H., Mullen G.R., Durden L.A. (2019). Ticks (Ixodida). Medical and Veterinary Entomology.

[8] Scott J.D., Pascoe E.L., Sajid M.S., Foley J.E. (2020). Detection of *Babesia odocoilei* in *Ixodes scapularis* ticks collected from songbirds in Ontario and Quebec, Canada. Pathogens.

[9] Scott J.D., Clark K.L., Foley J.E., Bierman B.C., Durden L.A. (2018). Far-reaching dispersal of *Borrelia burgdorferi* sensu lato-infected blacklegged ticks by migratory songbirds in Canada. Healthcare.

[10] Morshed M.G., Scott J.D., Fernando K., Beati L., Mazerolle D.F., Geddes G., Durden L.A. (2005). Migratory songbirds disperse ticks across Canada, and first isolation of the Lyme disease spirochete, *Borrelia burgdorferi*, from the avian tick, *Ixodes auritulus*. J. Parasitol..

[11] Scott J.D., Clark K.L., Foley J.E., Anderson J.F., Bierman B.C., Durden L.A. (2018). Extensive distribution of the Lyme disease bacterium, *Borrelia burgdorferi* sensu lato, in multiple tick species parasitizing avian and mammalian hosts across Canada. Healthcare.

[12] Reed K.D., Meece J.K., Henkel J.S., Shukla S.K. (2002). Birds, migration and emerging zoonoses: West Nile virus, Lyme disease, Influenza A and enteropathogens. Clin. Med. Res..

[13] Scott J.D., Clark K.L., Coble N.M., Ballantyne T.R. (2019). Presence of *Babesia odocoilei* and *Borrelia burgdorferi* sensu stricto in a tick and dual parasitism of *Amblyomma inornatum* and *Ixodes scapularis* on a bird in Canada. Healthcare.

[14] Scott J.D., Clark K.L., Coble N.M., Ballantyne T.R. (2019). Detection and transstadial passage of *Babesia* species and *Borrelia burgdorferi* sensu lato in ticks collected from avian and mammalian hosts in Canada. Healthcare.

[15] Hersh M.H., Ostfeld R.S., McHenry D.J., Tibbetts M., Brunner J.L., Killilea M.E., LoGiudice K., Schmidt K.A., Kessing F. (2014). Co-infection of blacklegged ticks with *Babesia microti* and *Borrelia burgdorferi* is higher than expected and acquired from small mammal hosts. PLoS ONE.

[16] Scott J.D., Sajid M.S., Pascoe E.L., Foley J.E. (2021). Detection of *Babesia odocoilei* in humans with babesiosis symptoms. Diagnostics.

[17] Emerson H.R., Wright W.T. (1968). The isolation of a *Babesia* in white-tailed deer. Bull. Wildl. Dis. Assoc..

[18] Emerson H.R., Wright W.T. (1970). Correction. J. Wildl. Dis..

[19] Holman P.J., Madeley J., Craig T.M., Allsopp B.A., Allsopp M.T., Petrini K.R., Waghela S.D., Wagner G.G. (2000). Antigenic, phenotypic and molecular characterization confirms *Babesia odocoilei* isolated from three cervids. J. Wildl. Dis..

[20] Eshoo M.W., Carolan H.E., Massire C., Chou D.M., Crowder C.D., Rounds M.A., Phillipson C.A., Schutzer S.E., Ecker D.J. (2015). Survey of *Ixodes pacificus* ticks in California reveals a diversity of microorganisms and a novel and widespread Anaplasmataceae species. PLoS ONE.

[21] Waldrup K.A., Kocan A.A., Barker R.W., Wagner G.G. (1990). Transmission of *Babesia odocoilei* in white-tailed deer (*Odocoileus virginianus*) by *Ixodes scapularis* (Acari: Ixodidae). J. Wildl. Dis..

[22] Hamer S.A., Roy P.L., Hickling G.J., Walker E.D., Foster E.S. (2007). Zoonotic pathogens in *Ixodes scapularis*, Michigan. Emerg. Infect. Dis..

[23] Steiner F.E., Pinger R.R., Vann C.N., Grindle N., Civitello D., Clay K., Fuqua C. (2008). Infection and co-infection rates of *Anaplasma phagocytophilum* variants, *Babesia* spp., *Borrelia burgdorferi*, and the rickettsial endosymbiont in *Ixodes scapularis* (Acari: Ixodidae) from sites in Indiana, Maine, Pennsylvania, and Wisconsin. J. Med. Entomol..

[24] Livengood J., Hutchinson M.L., Thirumalapura N., Tewari D. (2020). Detection of *Babesia*, *Borrelia*, *Anaplasma*, and *Rickettsia* spp. in adult black-legged ticks (*Ixodes scapularis*) from Pennsylvania, United States, with Luminex Multiplex Bead Assay. Vector Borne Zoonotic Dis..

[25] Scott J.D., Pascoe E.L., Sajid M.S., Foley J.E. (2021). Detection of *Babesia odocoilei* in *Ixodes scapularis* ticks collected in southern Ontario, Canada. Pathogens.

[26] Waldrup K.A., Kocan A.A., Qureshi T., Davis D.S., Baggett D., Wagner G.G. (1989). Serological prevalence and isolation of *Babesia odocoilei* among white-tailed deer (*Odocoileus virginianus*) in Texas and Oklahoma. J. Wildl. Dis..

[27] Perry B.D., Nichols D.K., Cullom E.S. (1985). *Babesia odocoilei* Emerson and Wright, 1970 in white-tailed deer, *Odocoileus virginianus* (Zimmermann), in Virginia. J. Wildl. Dis..

[28] Shock B.C., Moncayo A., Cohen S., Mitchell E.A., Williamson P.C., Lopez G., Garrison L.E., Yabsley M.J. (2014). Diversity of piroplasms detected in blood-fed and questing ticks from several states in the United States. Ticks Tick Borne Dis..

[29] Schoelkopf L., Hutchinson C.E., Bendele K.G., Goff W.L., Willette M., Rasmussen J.M., Holman P.J. (2005). New ruminant hosts and wider geographic range identified for *Babesia odocoilei* (Emerson and Wright 1970). J. Wildl. Dis..

[30] Smith R.P., Elias S.P., Borelli T.J., Missaghi B., York B.J., Kessler R.A., Lubelczyk C.B., Lacombe E.H., Hayes C.M., Coulter M.S. (2014). Human babesiosis, Maine, USA, 1995–2011. Emerg. Infect. Dis..

[31] Pattullo K.M., Wobeser G., Lockerbie B.P., Burgess H.J. (2013). *Babesia odocoilei* infection in a Saskatchewan elk (*Cervus elaphus canadensis*) herd. J. Vet. Diagn. Investig..

[32] Horowitz R.I. (2013). Lyme and Other Co-infections: Parasitic, Viral, and Fungal Infectivity. Why Can’t I Get Better?.

[33] Kinderlehrer D.A. (2021). Babesia. Recovery from Lyme disease: The Integrative Medicine Guide to Diagnosing and Treating Tick-Bore Illness.

[34] Scott J.D., Pascoe E.L., Sajid M.S., Foley J.E. (2020). Monitoring of nesting songbirds detects established population of blacklegged ticks and associated Lyme disease endemic area in Canada. Healthcare.

[35] Baneth G., Mathew J.S., Shkap V., Macintire D.K., Barta J.R., Ewing S.A. (2003). Canine hepatozoonosis: Two disease syndromes caused by separate *Hepatozoon* spp.. Trends Parasitol..

[36] Macintire D.K., Vincent-Johnson N., Dillon A.R., Blagburn B., Lindsay D., Whitley E.M., Banfield C. (1997). Hepatozoonosis in dogs: 22 cases (1989–1994). J. Am. Vet. Med. Assoc..

[37] Kaufman R.W., Sonenshine E.E., Roe R.M. (2013). Integument and ecdysis. Biology of Ticks.

[38] Richter D., Spielman A., Momar N., Matuschka F.-R. (2000). Competence of American Robins as reservoir hosts for Lyme disease spirochetes. Emerg. Infect. Dis..

[39] Babes V. (1888). Sur l’hémoglobinurie bactérienne du boeuf. C. R. Acad. Sci..

[40] Smith T., Kilborne F.L. (1983). Investigation into the Nature, Causation, and Prevention of Southern Cattle Fever.

[41] Škrabalo Z., Deanović Z. (1957). Piroplasmosis in man: Report on a case. Doc. Med. Geogr. Trop..

[42] Schetters T. (2019). Mechanisms involved in the persistence of *Babesia canis* infection in dogs. Pathogens.

[43] Gray J., Zintl A., Hildebrandt A., Hunfeld K.-P., Weiss L. (2010). Zoonotic babesiosis: Overview of the disease and novel aspects of pathogen identity. Ticks Tick Borne Dis..

[44] Scholtens R.G., Braff E.H., Healy R.R., Gleason N. (1968). A case of babesiosis in man in the United States. Am. J. Trop. Med. Hyg..

[45] Western K.A., Benson G.D., Gleason N.N., Healy G.R., Schultz M.G. (1970). Babesiosis in a Massachusetts resident. N. Engl. J. Med..

[46] Herwaldt B.L., Caccio S., Gherlinzoni F., Aspöck H., Siemenda S.B., Piccaluga P., Martinelli G., Edelhofer R., Hollenstain U., Poletti G. (2003). Molecular characterization of a non-*Babesia divergens* organism causing zoonotic babesiosis in Europe. Emerg. Infect. Dis..

[47] Shih C.M., Liu L.P., Chung W.C., Ong S.J., Wang C.C. (1997). Human babesiosis in Taiwan: Asymptomatic infection with a *Babesia microti*-like organism in Taiwanese woman. J. Clin. Microbiol..

[48] Kjemtrup A.M., Conrad P.A. (2000). Human babesiosis: An emerging tick-borne disease. Int. J. Parasitol..

[49] Herwaldt B.L., Persing D.H., Precigout E.A., Goff W.L., Mathiesen D.A., Taylor P.W., Eberhard M.L., Gorenflot A.F. (1996). A fatal case of babesiosis in Missouri: Identification of another piroplasm that infects humans. Ann. Intern. Med..

[50] Ord R.L., Lobo C.A. (2015). Human babesiosis: Pathogens, prevalence, diagnosis, and treatment. Cur. Clin Microbiol. Rep..

[51] Kim J.-Y., Cho S.-H., Joo H.-N., Tsuji M., Cho S.-R., Park I.-J., Chung G.-T., Ju J.-W., Cheun H.-I., Lee H.-W. (2007). First case of human babesiosis in Korea: Detection and characterization of a novel type of *Babesia* sp. (KO1) similar to ovine *Babesia*. J. Clin. Microbiol..

[52] Zintl A., Mulcahy G., Skerrett H.E., Taylor S.M., Gray J.S. (2003). *Babesia divergens*, bovine blood parasite of veterinary and zoonotic importance. Clin. Microbiol. Rev..

[53] Tonnetti L., Young C., Kessler D.A., Williamson P.C., Reik R., Proctor M.C., Brès V., Deisting B., Bakkour S., Schneider W. (2020). Transcription-mediated amplification blood donation screening for *Babesia*. Transfusion.

[54] Brennan M.B., Herwaldt B.L., Kazmierczak J.J., Weiss J.W., Klein C.L., Leith C.P., He R., Oberley M.J., Tonnetti. L., Wilkins P.P. (2016). Transmission of *Babesia microti* parasites by solid organ transplantation. Emerg. Infect. Dis..

[55] New D.L., Quinn J.B., Qureshi M.Z., Sigler S.J. (1997). Vertically transmitted babesiosis. J. Pediatr..

[56] Fox L.M., Winger S., Ahmed A., Arnold A., Chou J., Rhein L., Levy O. (2006). Neonatal babesiosis: Case report and review of the literature. Pediatr. Infect. Dis. J..

[57] Cornett J.K., Malhotra A., Hart D. (2012). Vertical transmission of babesiosis from a pregnant, splenectomized mother to her neonate. Infect. Dis. Clin. Pract..

[58] Iyer S., Goodman K. (2019). Congenital babesiosis from maternal exposure: A case report. J. Emerg. Med..

[59] Lavoie P.E., Lattner B.P., Duray P.H., Barbour A.G., Johnson P.C. (1987). Culture positive seronegative transplacental Lyme borreliosis infect morality. Arthritis Rheum..

[60] MacDonald A.B. (1986). Human fetal borreliosis, toxemia of pregnancy, and fetal death. Zentralbl. Bakteriol. Mikrobiol. Hyg. A..

[61] Trevisan G., Stinco G., Cinco M. (1997). Neonatal skin lesions due to spirochetal infection; A case of congenital Lyme borreliosis?. Int. J. Dermatol..

[62] Horowitz R.I. Lyme disease and pregnancy: Implication of chronic infection, PCR testing, and prenatal treatment. In Proceeding of the 16th International Scientific Conference on Lyme Disease & Other Tick-Borne Disorders.

[63] Gardner T., Remington J.S., Klein J.O. (2001). Lyme disease. Infectious Diseases of the Fetus and Newborn Infant.

[64] Eisen R.J., Eisen L., Beard C.B. (2016). Country-scale distribution of *Ixodes scapularis* and *Ixodes pacificus* (Acari: Ixodidae) in the continental United States. J. Med. Entomol..

[65] Penzhorn B.L., Oosthuizen M.C. (2020). *Babesia* species of domestic cats: Molecular characterization has opened Pandora’s box. Front. Vet. Sci..

[66] Keirans J.E., Clifford C.M. (1978). The genus *Ixodes* in the United States: A scanning electron microscope study and key to the adults. J. Med. Entomol..

[67] Keirans J.E., Hutcheson H.J., Durden L.A., Klompen J.S.H. (1996). *Ixodes* (*Ixodes*) *scapularis* (Acari: Ixodidae): Redescription of all active stages, distribution, hosts, geographical variation, and medical and veterinary importance. J. Med. Entomol..

[68] Durden L.A., Keirans J.E. (1996). Nymphs of the Genus Ixodes (Acari: Ixodidae) of the United States: Taxonomy, Identification Key, Distribution, Hosts, and Medical Veterinary Importance, Monographs.

[69] Foley J., Tinoco-Gracia L., Rodriguez-Lomelf M., Estrada-Guzmán J., Fierro M., Mattar-Lopez E., Peterson A., Pascoe E., Gonzalez Y., Hori-Oshima S. (2019). Unbiased assessment of abundance of *Rhipicephalus sanguineus* sensu lato ticks, canine exposure to spotted fever group *Rickettsia*, risk factor in Mexicali, México. Am. J. Trop. Med. Hyg..

[70] Barbour A.G., Bunikis J., Tranvinsky B., Hoen A.B., Diuk-Wasser M.A., Fish D., Tsao J.I. (2009). Niche partitioning of *Borrelia burgdorferi* and *Borrelia miyamotoi* in the same tick vector and mammalian reservoir species. Am. J. Trop. Med. Hyg..

[71] Drazenovich N., Foley J., Brown R.N. (2006). Use of real-time quantitative PCR targeting the *msp2* protein gene to identify cryptic *Anaplasma phagocytophilum* infections in wildlife and domestic animals. Vector Borne Zoonotic Dis..

[72] Black W.C., Piesman J. (1994). Phylogeny of hard- and soft-tick taxa (Acari: Ixodidae) based on mitochondrial 16S rDNA sequences. Proc. Natl. Acad. Sci. USA.

[73] Beati L., Keirans J.E. (2001). Analysis of the systematic relationships among ticks of the genera *Rhipicephalus* and *Boophilus* (Acari: Ixodidae) based on mitochondrial 12 ribosomal DNA gene sequences and morphological characters. J. Parasitol..

