# Expression of Concern: Scott, J.D.; Pesapane, R.R. Detection of *Anaplasma phagocytophilum*, *Babesia odocoilei*, *Babesia* sp., *Borrelia burgdorferi* Sensu Lato, and *Hepatozoon canis* in *Ixodes scapularis* Ticks Collected in Eastern Canada. *Pathogens* 2021, *10*, 1265

**DOI:** 10.3390/pathogens15040369

**Published:** 2026-03-31

**Authors:** Pathogens Editorial Office

**Affiliations:** MDPI AG, St. Alban-Anlage 66, 4052 Basel, Switzerland; pathogens@mdpi.com

With this notice, the *Pathogens* Editorial Office alerts the readers to concerns related to this article [1]. Following publication, concerns were raised to the Editorial Office regarding the attribution of supplementary contextual information included in Section 3.10 of this article [1]. The authors and their affiliated institution have been contacted to assist with the investigation. Upon the conclusion of the investigation, the Editorial Office, with the involvement of the Editor-in-Chief, has decided to issue this Expression of Concern.
